# Mitochondrial *COI* and 16sRNA Evidence for a Single Species Hypothesis of *E*. *vitis*, *J*. *formosana* and *E*. *onukii* in East Asia

**DOI:** 10.1371/journal.pone.0115259

**Published:** 2014-12-15

**Authors:** Jian-Yu Fu, Bao-Yu Han, Qiang Xiao

**Affiliations:** 1 Key Laboratory of Tea Plants Biology and Resources Utilization of Agriculture Ministry, Tea Research Institute, Chinese Academy of Agricultural Sciences, Hangzhou, 310008, PR China; 2 Graduate School of Chinese Academy of Agricultural Sciences, Beijing, 100081, PR China; 3 Zhejiang Provincial Key Laboratory of Biometrology and Inspection & Quarantine, College of Life Sciences of China Jiliang University, Hangzhou, 310018, PR China; St. Petersburg Pasteur Institute, Russian Federation

## Abstract

Tea green leafhopper is one of the most damaging tea pests in main tea production regions of East Asia. For lack of recognized morphological characters, the dominant species of tea green leafhoppers in Mainland China, Taiwan and Japan have always been named as *Empoasca vitis* Göthe, *Jacobiasca formosana* Paoli and *Empoasca onukii* MATSUDA, respectively. Furthermore, nothing is known about the genetic relationships among them. In this study, we collected six populations from Mainland China, four populations from Japan and one population from Taiwan, and examined the genetic distances in the *COI* and 16sRNA regions of mtDNA among them. The results showed that the genetic distances based on single gene or the combined sequences among eleven leafhopper populations were 0.3–1.2%, which were all less than the species boundary of 2%. Moreover, there were at least two haplotypes shared by two distinct populations from different regions. The phylogenetic analysis based on single gene or combined sets also supported that tea green leafhoppers from Mainland China, Taiwan and Japan were closely related to each other, and there were at least two specimens from different regions clustered ahead of those from the same region. Therefore, we propose that the view of recognizing the dominant species of tea green leafhoppers in three adjacent tea production regions of East Asia as different species is unreliable or questionable and suggest that they are a single species.

## Background

Tea, *Camellia sinensis* (L.) O. Kuntze, originated in China and is now grown in almost sixty countries. In East Asia, Mainland China, Japan and Taiwan are three main tea production regions. The tea plantation is a very stable ecosystem for plenty of insects to colonize. Globally, 1031 arthropod species are associated with tea plants, which are always the most damaging constraint, causing on average a 5% to 55% yield loss [1], [2]. The distribution of insect pests in tea plantations is related to the regional climate, planting history, tea varieties, cultivation management, pest management model, tea production characteristic, as well as other factors.

Tea green leafhopper is one of the most important pests in East Asia. It belongs to Hemiptera, Jassidioea, Cicadellidae, which is one of the most diverse families of terrestrial organisms [3], [4]. As the most dominant pest in Chinese tea plantations, tea green leafhopper has been attracting a lot of attention from both Chinese governments and farmers [5]. The adult tea green leafhopper is about 3 mm long and green with transparent colourless wings. There are four nymphal instars that look like the adults with no wings. Both adults and nymphs of tea leafhopper pierce and suck the sap of tender tea shoots, which results in leaf edge yellowing, leaf tine curling, vein reddening, tea shoot growing slowly or stagnating, and even leaf edge and tine hennaing and withering [6]. In most regions of Mainland and Taiwan, there are 9 to 15 overlapping generations of tea green leafhoppers per year, and it causes, on average 15–20% annual yield loss and deterioration of commercial tea quality [7]. In Japan, tea green leafhopper occurs not so frequently as China with 5 to 8 generations a year.

The leafhopper is an extraordinarily large group with a wide range of host plants. Over 25,000 species of Cicadellidae are described and new leafhopper species are being discovered almost every day, but few useful morphological characteristics are developed to understand the relationships between leafhopper species and ecological factors for diversification [4], [8]. Until now, it has been clear that there is just one dominant species of tea green leafhopper in these three regions, respectively [9]–[11]. However, for lack of recognized morphological characters and compared research communications, the dominant species of tea green leafhoppers in Mainland China, Taiwan and Japan are always named as different species; *Empoasca vitis* Göthe, *Jacobiasca formosana* Paoli and *Empoasca onukii* MATSUDA, respectively [5], [9], [11].

Mitochondrial DNA (mtDNA) is widely used in taxonomy and systematics to elucidate the phylogenetic relationships of insects [12], [13], especially among some sibling species and cryptic species which are difficult to distinguish solely by morphology [14], [15]. The mtDNA sequence was first employed to find relationships among species within a genus of leafhoppers by Dietrich in 1997 [4]. Since then, more and more mtDNA markers were developed to combine with morphological characters to diagnose the systematics of Cicadellidae and the co-evolution of leafhoppers and their bacterial symbionts. The mtDNA sequence information can be useful for detecting differences among putative insect species [16] or well-resolved biotypes [17]–[19]. Cytochrome Oxidase I (*COI*), is successfully used for leafhopper species identification, phylogeographical and phylogeny studies of sibling species, thus being well-suited for examining intra- and inter-specific variation at both the species and genus level [20]. The 16sRNA is already used for species-level phylogenetic studies of leafhoppers (Cicadellidae) and especially the closely related species with discrete informative morphological characters [4].

The tea plant spread from Mainland China to neighbouring regions such as Japan and Taiwan. Tea green leafhoppers, important insect pests in tea plantations, which have identical morphological characteristics and biological habits, are differently named in Mainland China, Taiwan and Japan. Furthermore, nothing is known about tea green leafhoppers evolution relationship in these regions. Here, we present the hypothesis that they might be a single species. In this study, we surveyed the mtDNA sequences of tea leafhoppers among eleven populations in Mainland China, Taiwan and Japan, to understand the genetic divergences, phylogenetic relationship, to prove the hypothesis that the tea green leafhopper species of *E*. *vitis*, *J*. *formosana* and *E*. *onukii* are a single species.

## Materials and Methods

### Ethics Statement

No specific permits were required for this study. The tea green leafhoppers are agricultural pests and are not endangered or protected species. All samples were collected in open tea plantations and not from any national parks or protected areas.

### Sample collection and preparation

**Figure 1:**
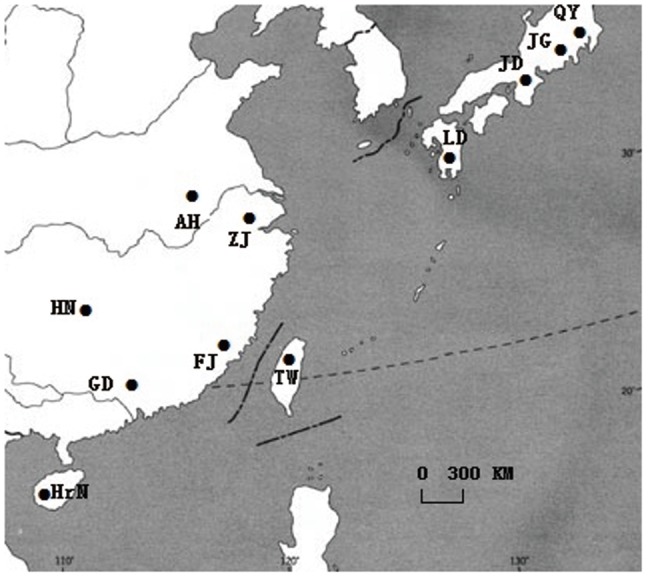
The distribution map of eleven tea green leafhopper populations. Six populations were sampled from Anhui (AH), Zhejiang (ZJ), Hunan (HN), Fujian (FJ), Guangdong (GD) and Hainan (HrN) in main tea production regions of Mainland China. One population was collected from Taiwan, and four populations were collected from Shizuoka (JG), Kyoto (JD), Saitama (QY) and Kagoshima (LD) in Japan.

A relative number of collection sites were chosen from each area (Fig. 1). To mitigate the possibility of collecting other leafhopper species that migrate from neighbouring habitats into the tea plantations to survive during the cold season, we collected all the specimens in the seasons of tea leafhopper outbreak from 2005 to 2010. Tea leafhopper specimens were collected by sweep netting from six main tea producing provinces of Zhejiang, Anhui, Guangdong, Fujian, Hainan and Hunan in Mainland China, four main tea production areas of Shizuoka, Kyoto, Saitama and Kagoshima in Japan, and Taoyuan in Taiwan (Table 1). The peach green leafhopper *Empoasca flavescens* was as the out-group and collected at a riverside open park, which was absolutely isolated from tea plantations, in downtown Hangzhou of Zhejiang province. All insects were rinsed in 70% ethanol three times, and stored at −80°C in 100% ethanol until DNA was extracted. All tea green leafhoppers were first identified under a stereo microscope following the keysincluding head and thorax, forewing, wing, and genitalia characters were used for diagnosing specimens before being used [9]. The insects and DNA samples were deposited in the Tea Research Institute, Chinese Academy of Agricultural Sciences, CAAS, Hangzhou, China.

**Table 1:** The collecting information and bioinformatics for leafhopper specimens.

Leafhopper specimens	Collecting locality	Longitude°(E)/Latitude°(N)	Collecting date (M/Y)	Population number	Accession number/Haplotypes	
**Ingroup Population**					***COI*** **/73**	**16sRNA/67**
*Empoasca vitis* (Göthe)	Mainland China, (Anhui, AH)	119.08/30.58	6/2005	12	KC172464–KC172527	KC172397–KC172456, KC172463
*Empoasca vitis* (Göthe)	Mainland China, (Fujian, FJ)	119.13/26.05	8/2004	14		
*Empoasca vitis* (Göthe)	Mainland China, (Guangdong, GD)	113.24/24.12	5/2008	13		
*Empoasca vitis* (Göthe)	Mainland China, (Hainan, HrN)	109.29/19.30	9/2005	14		
*Empoasca vitis* (Göthe)	Mainland China, (Hunan, HN)	113.26/28.27	6/2009	6		
*Empoasca vitis* (Göthe)	Mainland China, (Zhejiang, ZJ)	120.10/30.16	7/2004	14		
*Empoasca onukii* MATSUDA	Japan, (Shizuoka, JG)	138.38/34.98	9/2008	15		
*Empoasca onukii* MATSUDA	Japan, (Kyoto, JD)	135.46/35.10	10/2008	12		
*Empoasca onukii* MATSUDA	Japan, (Saitama, QY)	139.65/35.86	9/2008	14		
*Empoasca onukii* MATSUDA	Japan, (Kagoshima, LD)	130.33/31.35	9/2008	14		
*Jacobiasca formosana* (Paoli)	Taiwan, (Taoyuan, TW)	121.18/24.59	6/2006	13		
**Outgroup species**						
*Empoasca flavescens* (Fabricius)	China, (Zhejiang, Hangzhou)	120.12/30.16	7/2010	9	KC191708–KC191716	KC172457–KC172462

### DNA extraction

Total DNA was extracted from a single insect body after it air-dried for 20 min. The specimen tissue was first ground with a flame-sealed pipette tip in a 1.5 ml microcentrifuge tube, and then incubated at 55°C in 0.5 ml lysis buffer containing 100 mM Tris-HCl, 5 mM EDTA, 200 mM NaCl, 0.5% SDS, and 100 µg/ml proteinase K for 5 hours. The lysis products were treated with a standard phenol-chloroform-isoamyl alcohol (25∶24∶1) method, and DNA was precipitated from the supernatant with two volumes of cold ethanol, centrifuged, washed, dried and dissolved in 100 µl deionized and sterilized water, and then stored at −80°C until use.

### PCR amplification and sequencing

Polymerase chain reactions were carried out on an ABI Veriti thermocycler in 50 µl total volumes containing 5 ng DNA, 1.5 mM MgCl_2_, 0.25 mM dNTPs, 0.2 µM of each primer and 0.5 Units *taq* NEB DNA polymerase. We amplified the *COI* genes with primers C1-J-2195: 5′-TTGATTTTTTGGTCAYCCWGAAGT-3′ and TL2-N-3014: 5′-TTCATTGCACTAATCTGCCATACTA-3′ [21]. The 16sRNA fragments were obtained by primers LR-J-12887: 5′-CCGGTYTGAACTCARATCAWGT-3′ and LR-N-13398: 5′-CTGTTTAWCAAAAACATTTC-3′ [4]. The PCR program was denatured 4 min at 94°C, followed by 30 cycles of 94°C for 30 sec, 55°C for 30 sec, 72°C for 1 min and extension at 72°C for 10 min. The PCR products were detected by electrophoresis on a 1.2% agarose gel stained with ethidium bromide, purified with the Gel DNA extraction kit (Axygen), introduced into pGEM T-easy vector (Promega) and sequenced bidirectionally by an ABI 3730 automated sequencer with the universal primers M13F: 5′-CGCCAGGGTTTTCCCAGTCACGAC-3′ and M13R: 5′-AGCGGATAACAATTTCACACAGGA-3′. At least two copies of each clone were sequenced simultaneously.

### Phylogenetic analysis and haplotype network

Both sense and antisense sequences were automatically aligned and assembled by DNAMAN 4.0 software, and the single consensus sequence was obtained by manual splicing. All these mtDNA sequences were further confirmed by checking if they could be appropriately translated into protein using invertebrate mitochondrial codes by MEGA 6.0 program [22]. The *COI*, 16sRNA and their combined sequences were aligned by Clustal Omega software online (http://www.ebi.ac.uk/Tools/msa/clustalo/), the alignments were downloaded and converted to compatible format by MEGA 6.0 for analysis below.

All these analyses were done based on both single gene and the combined sequences. The sequence polymorphism level including nucleotide diversity (*Pi*), haplotype diversity (*Hd*), and the number of segregating sites (*S*) was calculated by DnaSP 5.0. Genetic distances between gene pairs were calculated using the Kimura 2-parameter (K2-P) model via MEGA 6.0. Neutrality tests of Tajima's D, Fu and Li's D, Fu and Li's F were performed by DnaSP 5.0 [23]. The amount of variation within a population and among populations within a region was calculated by the hierarchical analysis of molecular variance (AMOVA) framework carried out using ARLEQUIN 3.11, and significant difference was tested by a nonparametric permutation procedure with 1000 permutations. The patterns of mitochondrial DNA polymorphism and evolution among *E*. *vitis*, *J*. *formosana* and *E*. *onukii* were also analyzed. Population differentiation was also quantified with non hierarchical analysis of molecular variance by estimating fixation index (*Fst*) of Mainland China-Japan, Japan-Taiwan, and Mainland-Taiwan populations.

The phylogenetic trees were constructed based on single gene and combined *COI* and 16sRNA by two methods of Maximum likelihood (ML) and Bayesian (BI). ML analysis was performed by RAxML online (http://www.trex.uqam.ca/index.php?action=raxml) and BI analysis was performed using GTR+I+G model via MrBayes 3.2.2. The BI trees were visualized and produced using FigTree software version 1.4.2, and only three BI trees for single gene and combined sets are presented here. The haplotypes of combined sequences were analyzed using the median-joining algorithm, and the network reconstruction were manual performed with Network 4.6.1.0 [24].

## Results

### Morphological examination

The *E. onukii* was nominated from leafhoppers in tea plantation by Matsuda in 1952 [25]. The dominant species of *E. vitis* and *J. formosana* in tea plantations of China Mainland and Taiwan were both named according to nominated species from other host plants. *E. vitis* was first named in 1875, and it was recognized as the dominant tea leafhopper species of China Mainland in 1988 [9], [26]; *J. formosana* was named in 1932, and it was identified as the dominant species in tea plantations of Taiwan [5], [27]. Utilizing the main, generally accepted, keys for tea green leafhopper [9], [25], we found no difference in these eleven population specimens.

### Sequences analysis

A total of 149 partial *COI* and 16sRNA fragments were separately obtained and sequenced, of which 140 were from eleven leafhopper populations and nine from the out-group *E. flavescens*. The mtDNA *COI* fragment was 773 bp long with 208 polymorphic sites and 175 parsimony informative sites, and the A+T content was about 71%. In total, we obtained 64 haplotypes and the haplotype diversity index (*Hd*) was 0.99. As well, all nine *COI* genes from peach green leafhoppers are haplotypes. The 16sRNA gene was 535 bp long, which contained 129 polymorphic sites and 85 parsimony informative sites, and the A+T content was about 75%. We detected 61 haplotypes for 16sRNA in tea green leafhopper populations and the haplotype diversity was 0.95, and nine out-group sequences are all haplotypes. Sequences of 64 haplotypes for *COI* and 61 haplotypes for 16sRNA of tea green leafhoppers were finally deposited in GenBank (*COI* Accession Nos. KC172464-KC172527 and 16sRNA Accession Nos. KC172397-KC172456, KC172463), and the genes for out-group *E. flavescens* were also deposited respectively (Accession Nos. KC191708-KC191716 and KC172457-KC172462) (Table 1).

We also analyzed the combined mtDNA *COI* and 16sRNA genes simultaneously; 112 haplotypes were observed in 140 tea green leafhoppers specimens, and the haplotype diversity was high, more than 0.99. There were only eight haplotypes that contained more than two samples, of which FJ13 was the largest one and shared by nine samples of GD2, GD12, GD14, HrN6, HrN8, HrN12, TW4, TW10 and LD16 from all three regions of Mainland China, Taiwan and Japan. The haplotype of FJ2 was shared by five samples of FJ10, JG5, JG15, QY12 and QY17 from two regions of Mainland China and Japan. Three haplotypes of AH3, GD1 and AH11 were shared by two, two and seven samples from Mainland China respectively. The haplotypes of JD17, JG12 and QY8 were shared by two, three and three samples from Japan respectively. The other 104 haplotypes were all unique ones and the nine combined sets from *E. flavescens* were all unique haplotypes, too. The patterns of mitochondrial DNA polymorphism and evolution among the three leafhopper species are shown in Table 2. The values detected in *E*. *vitis* and *E*. *onukii* were much lower than those of *J*. *formosana*. Furthermore, the tested values for Tajima's D, Fu and Li's D and Fu and Li's F in *E*. *vitis* and *E*. *onukii* were all obviously less than those in *J*. *formosana*.

**Table 2:** Comparison of combined *COI* and 16sRNA sequences evolution of *E*. *vitis*, *J*. *formosana* and *E*. *onukii*.

Species	n	h	Hd	S	Pi	D	Tajima's D	Fu and Li's D	Fu and Li's F
*E*. *vitis*	74	59	0.983	94	0.00565	−2.10751*	−2.16308**	−4.51381**	−4.25515**
*E*. *onukii*	56	48	0.992	58	0.00368	−2.19429***	−2.71467***	−4.19148**	−4.11439**
*J*. *formosana*	10	9	0.978	24	0.00644	−1.280251	−0.22352	0.13177	0.04879

The letters n, h, Hd, S, Pi and D are the no. of tea green leafhopper samples, no. of mitochondrial DNA haplotype, no. of segregating sites, haplotype diversity, nucleotide diversity and genetic divergence, respectively. *: *P*<0.05; **: *P*<0.02; ***: *P*<0.01.

### Genetic distance

The genetic divergences for *COI*, 16sRNA and combined sequences among 64, 61 and 112 leafhopper haplotypes of eleven populations were 0.3%–1.2%, 0.3%–0.6% and 0.3%–0.9%, and the average values were 0.7%, 0.8% and 0.6% respectively. All the genetic distances were at low levels and those for combined datasets are shown in Table 3. All the pairwise genetic distances between each two samples of tea green leafhopper and out-group *E. flavescens* were 31.3%–31.6%, which was 40-fold greater than those of in-group. The mean distances within each leafhopper population were also calculated and shown in Table 4 based on *COI*, 16sRNA and combined sets, which were also at much lower levels with the values of 0.2%–0.9%.

**Table 3:** Mean distance among tea green leafhopper populations based on combined datasets of *COI* and 16sRNA.

Population	AH	FJ	GD	HN	HrN	ZJ	TW	JG	JD	QY	LD	PL
AH												
FJ	0.005											
GD	0.006	0.005										
HN	0.005	0.005	0.005									
HrN	0.006	0.005	0.005	0.005								
ZJ	0.006	0.005	0.005	0.005	0.005							
TW	0.008	0.007	0.007	0.007	0.007	0.007						
JG	0.009	0.007	0.008	0.008	0.007	0.008	0.008					
JD	0.009	0.008	0.008	0.008	0.008	0.008	0.008	0.003				
QY	0.009	0.008	0.008	0.008	0.008	0.008	0.008	0.003	0.003			
LD	0.008	0.007	0.007	0.007	0.007	0.007	0.008	0.004	0.005	0.004		
PL	0.315	0.313	0.314	0.314	0.314	0.315	0.317	0.316	0.316	0.316	0.316	

**Table 4:** Mean distance within tea green leafhopper populations based on *COI*, 16sRNA and combined datasets.

Population	*COI*	16sRNA	*COI*+16sRNA
AH	0.0072	0.0054	0.0061
FJ	0.0085	0.0044	0.0048
GD	0.0059	0.0043	0.0054
HN	0.0061	0.0029	0.0044
HrN	0.0078	0.0026	0.0053
ZJ	0.0059	0.0048	0.0052
JG	0.0022	0.0057	0.0026
JD	0.0051	0.0046	0.0038
QY	0.0026	0.0059	0.0026
LD	0.0042	0.0058	0.0053
TW	0.0070	0.0059	0.0084

### Phylogenetic analysis and haplotype network

**Figure 2:**
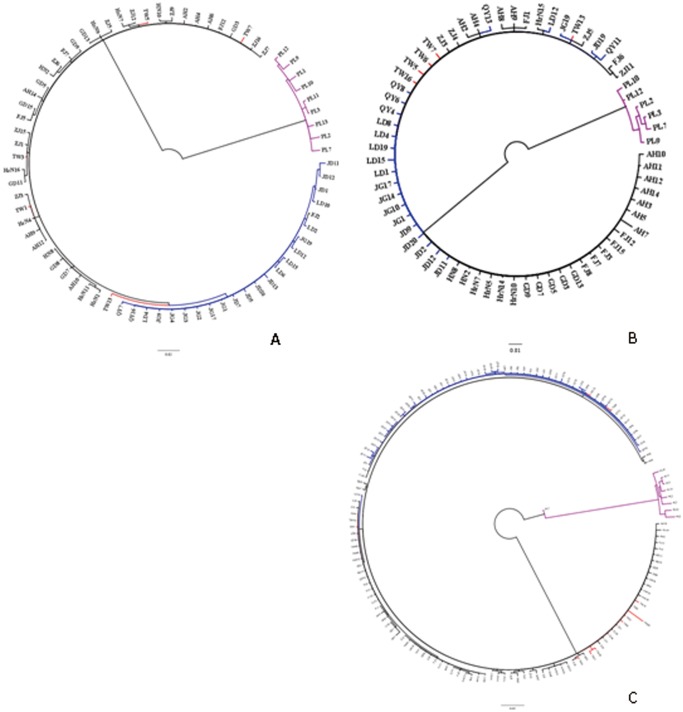
The BI phylogenetic trees for *COI* (A), 16sRNA (B) and combined sequences (C) of eleven leafhopper populations from Mainland China, Taiwan and Japan. The samples from Mainland China, Japan and Taiwan were shown with black, blue and red colour, respectively; and the out-group of *E. flavescens* was shown with purple colour. These analyses involved 73, 67 and 149 nucleotide sequences including the out-group, respectively. Evolutionary analyses were conducted using MrBayes 3.2.2.

**Figure 3:**
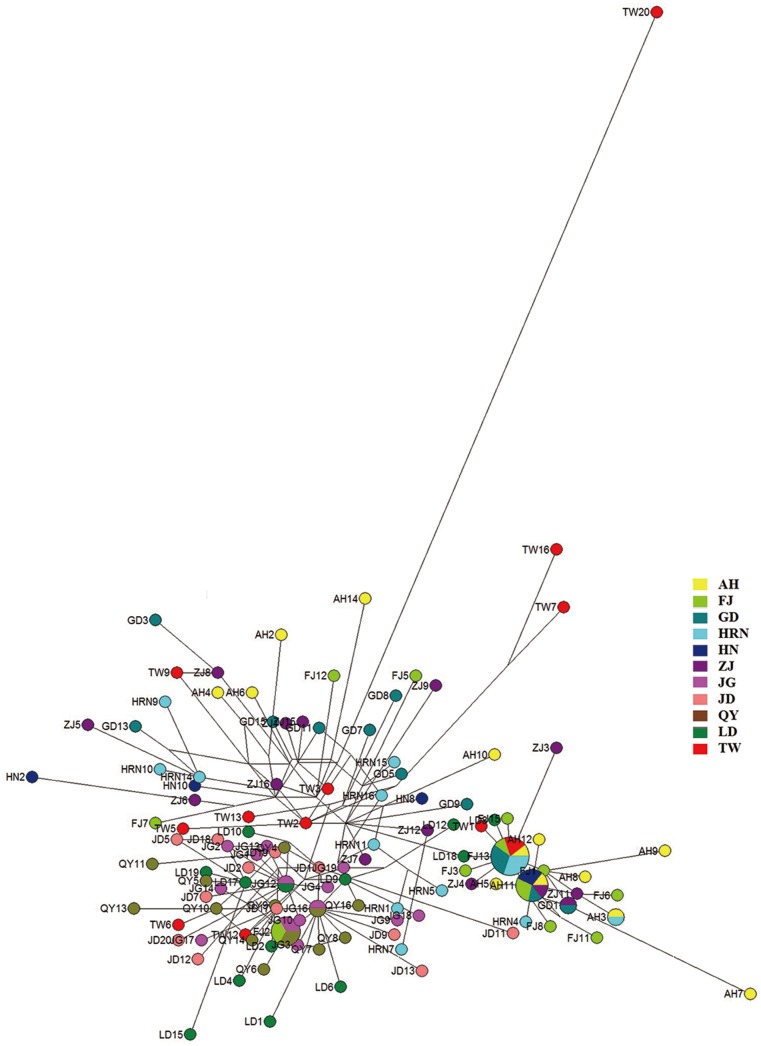
The haplotype network for combined *COI* and 16sRNA sequences of leafhoppers. The populations were presented with different colours and the circle area showed the proportional of haplotype number.

In the both BI and ML trees for *COI* gene, although most Japanese haplotypes clustered closely, the FJ2 haplotype from Mainland China first clustered with LD2 from Japan, and then clustered with other Japanese haplotypes together. Furthermore, the five Taiwan haplotypes always clustered first with haplotypes from at least three populations in Mainland China. To the both two trees for 16sRNA genes, no prior clustering pattern was found for haplotypes from the same regions. In the BI and ML trees for combined sequences, three samples LD8, LD16 and LD18 form Japan clustered prior with many samples from Mainland China, and two samples FJ2, FJ10 from Mainland China and two samples TW6, TW12 from Taiwan clustered prior with some samples from Japan. The BI trees for haplotypes of *COI* and 16sRNA, and the BI tree for combined sequences (S1, S2, S3 Figures) are only presented here (Fig. 2). Summarizing these phylogenetic trees, we also found that the leafhopper samples from Taiwan were relatively closer to Mainland China than those from Japan. The haplotype network that was reconstructed based on combined sequences showed no obvious divergent group (Fig. 3). The haplotypes FJ13, AH11 and FJ2 were three dominant haplotypes. FJ13 occurred in three populations of Mainland China, one population of Taiwan and one population of Japan. AH11 distributed in four populations from Mainland China, and FJ2 distributed in one population from Mainland China and two populations from Japan.

## Discussion

Tea plant spread from Mainland China to Japan and Taiwan along with the insect fauna. Tea green leafhoppers in the three regions have consistent morphological characters and biological habits [1], [6], [9], [11], but they are recognized as different species. Previously, we tested the 28S D2–D3 segments of genomic DNA among these leafhopper populations, and found the genetic divergences were much less than 0.1%. We had also tried some other mitochondrial genes to look for more helpful markers for genetic relationship studies; finally we found that only *COI* and 16sRNA genes could be stably amplified in all leafhopper DNA samples. Furthermore, we have investigated twelve populations of tea green leafhopper in China using the 16sRNA gene by another pair of primers and found the genetic distances among them were all less than 0.8% [28]. In a word, these two mitochondrial genes had been proved useful and accepted in leafhoppers diagnosis [21], [29], [30], and their sequences were successfully obtained, so the *COI* and 16sRNA fragments were selected in this study.

It has been proved that the genetic distance for insect species boundary was 2% [31]. All eleven populations from Mainland China, Taiwan and Japan showed such close relationships that the genetic distances for single gene and combined sequences were both less than 1.2%, and the mean values were less than 0.8%, which were below the criterion of 2% for insect species differentiation. These genetic relationship results support the hypothesis that *E*. *vitis*, *J*. *formosana* and *E*. *onukii* are a single tea green leafhopper species. On the whole, the genetic distances between each two populations from Mainland and Taiwan were 0.5–0.8%, while the genetic distances among populations from either Mainland or Taiwan with populations from Japan were 0.7–0.9%. It revealed that the genetic relationship between *E*. *vitis* and *J*. *formosana* was closer than either of them with *E*. *onukii*, and all of which were below species level.

Both the single gene and combined datasets showed high haplotype diversity (*Hd*) greater than 0.95, which implied a high diversity in tea green leafhoppers. In addition, at least one haplotype was shared together by leafhoppers from Mainland China, Taiwan and Japan. For the *COI*, 16sRNA and combined sequences, the haplotypes of FJ2 and AH3, QY14, LD6, FJ7 and FJ13, and FJ13 were shared by *E*. *vitis*, *J*. *formosana* and *E*. *onukii*, respectively. The ubiquitous shared haplotypes indicated that tea green leafhoppers from three regions were not distinct clades and they might be a single species at evolution level.

All the ML and BI trees based on the same genes were consistent and; therefore, only three BI trees were presented here. The trees, whether for single gene or combined sequences, showed that at least two samples from different regions clustered prior to some samples from one region. Indicating that the three tea green leafhopper species had no strict distinct boundary, therefore they might be a single species, according to their mutual phylogenetic relationships. Furthermore, all of the leafhopper samples from Taiwan showed a prior cluster with samples from Mainland China than those from Japan. This result indicated the obvious relationships below species level among tea green leafhoppers from East Asia, and *J*. *formosana* had a closer relationship with *E*. *vitis* than *E*. *onukii*. Although both Japan and Taiwan learned tea from Mainland China, Japan grew tea plants one thousand years earlier than Taiwan, which led to a relatively long-term isolation. Moreover, the climate of Taiwan was more coincident with Mainland compared with that of Japan. These might be the two most possible reasons for *E*. *vitis* being much closer to *J*. *formosana* than to *E*. *onukii* based upon genetic distances and phylogenetic trees.

In summary, we first conducted genetic distances and phylogenetic relationships analysis of the *COI* and 16sRNA regions of mtDNA for tea green leafhopper dominant species in East Asia. Both the results showed that within *E*. *vitis*, *J*. *formosana* and *E*. *onukii* there existed small genetic differences that were still below species level, which supported the hypothesis of a single species. However, further research is needed such as the creation of useful morphological keys, as well as cross-breeding test to understand genetic relationships of tea green leafhoppers on a large scale.

## Supporting Information

S1 Figure **The BI phylogenetic tree for** ***COI*** **of eleven leafhopper populations from Mainland China, Taiwan and Japan.**
(EPS)

S2 Figure **The BI phylogenetic tree for 16sRNA of eleven leafhopper populations from Mainland China, Taiwan and Japan.**
(EPS)

S3 Figure **The BI phylogenetic tree for combined sequences of eleven leafhopper populations from Mainland China, Taiwan and Japan.**
(EPS)
